# Food Supply Chain and Business Model Innovation

**DOI:** 10.3390/foods9020132

**Published:** 2020-01-27

**Authors:** Saeed Nosratabadi, Amirhosein Mosavi, Zoltan Lakner

**Affiliations:** 1 Doctoral School of Management and Business Administration, Szent Istvan University, 2100 Godollo, Hungary; saeed.nosratabadi@phd.uni-szie.hu; 2 Institute of Research and Development, Duy Tan University, Da Nang 550000, Vietnam; 3 Department of Mathematics and Informatics, J. Selye University, 94501 Komarno, Slovakia; 4 Faculty of Health, Queensland University of Technology, 130 Victoria Park Road, Queensland 4059, Australia; 5 Department of Food Economics, Faculty of Food Science, Szent Istvan University, Villanyi str. 29-43, 1118 Budapest, Hungary; lakner.zoltan@etk.szie.hu

**Keywords:** business models, business model innovation, food supply chain, logistics, food security, literature review, supply chain management, sustainable development, PRISMA, systematic review

## Abstract

This paper investigates the contribution of business model innovations in the advancement of novel food supply chains. Through a systematic literature review, the notable business model innovations in the food industry are identified, surveyed, and evaluated. Findings reveal that the innovations in value proposition, value creation processes, and value delivery processes of business models are the successful strategies proposed in food industry. It is further disclosed that rural female entrepreneurs, social movements, and also urban conditions are the most important driving forces causing farmers to reconsider their business models. In addition, the new technologies and environmental factors are the secondary contributors in business model innovation for the food processors. It is concluded that digitalization has disruptively changed the food distributor models. E-commerce models and Internet-of-Things are reported as the essential factors causing retailers to innovate their business models. Furthermore, consumption demand and product quality are two main factors affecting the business models of all the firms operating in the food supply chain regardless of their positions in the chain. The findings of the current study provide an insight into the food industry to design a sustainable business model to bridge the gap between food supply and food demand.

## 1. Introduction

It is estimated that the world population would reach to three billion by 2050 [1]. Such dramatic rising population consequently results in increasing the food demand exponentially. On the other hand, it has been revealed that that the energy consumed per person increased from 2250 kcal (9400 KJ) in the 1960s to 2880 kcal (12,000 KJ) in 2015 [2]. Despite the acceptable performance of global food system in supplying food and decreasing the numbers of undernourished people, one in eight people were suffering from severe undernourishment in 2014 [3] and 815 million people in 2018 [4]. In addition to the demand side, the research shows that the food supply is facing serious problems due to climate changes. Drought, rising temperatures, changes in precipitation regimes, increase of CO_2_ levels are named the most critical issues influencing the yields of agricultural production, and these issues are expected to exacerbate in the next 50 years [5]. Such changes subsequently result in socioeconomic factors such as the increase of the prices [6]. Hence, to meet this steadily increasing food demand, the current food supply chain system and activities should be reconsidered.

The food supply chain (FSC) consists of a chain of activities elaborating how a product is produced and delivered to the final consumers. At each stage of the chain, value or values are added to the product by each player of the FSC (i.e., farmers, processors, distributors, and retailers). Therefore, along with the supply chain, there is a chain, so-called value chain, explaining the value/values added to a product in each step. In other words, numerous actors perform in each stage of the FSC to produce the final product from raw material and deliver it to the final consumers. Each of the entities have their objectives which may be contrasted with the other actors’ as the activities of each entity influence the performance of the whole supply chain [7]. The concept of business model provides the ability to design and analyze the value a business is offering and delivering to its customers [8]. The business model explains the position of a business in the value chain [9]. All the FSC actors have their own business models and they try to do their best to design elegantly and accurately their business models to increase competitiveness. Moreover, social [10], economic, and environmental factors [11] affect the design of business models of businesses in the food supply chain. Therefore, survival in the FSC is hard to manage [12] and it depends on the uniqueness of the business model.

Hence, analyzing the business model of all FSC players can provide the answers to many questions related to the food supply. Besides, any action to increase the food supply for meeting the future demand for food can be related to the business model of the FSC players. Thus, the main objective of the current study is to provide an insight illustrating how business models and innovations in business models contribute to businesses in different parts of the FSC to bridge the gap between food supply and food demand for future generations. To do so, a systematic literature review was conducted to find current solutions that are considered to optimize the production and deliver healthy foods to the consumers. Following, the methodology applied in this study is elaborated in detail, and after that, the findings are provided. It is worth mentioning that to provide a better understanding of the concepts used in this study, such as the FSC and business model strategies, they are defined and explained in advance. Ultimately, the discussion, contributions, and the possible implementation of the findings is provided.

## 2. Food Supply Chain

The food supply chain (FSC) comprises several stages in which food travels from the farmers to the final consumers [1]. In other words, a network of different actors in each stage of the FSC produces and delivers a final product to meet final customers’ needs. Much research is conducted to investigate and analyze the FSC, while the general consensus is that the main FSC actors are farmers, processors, distributors, retailer, and consumers (e.g., [7,13]). In a such FSC, the farmers harvest the initial production, processors produce the final products and package them, distributors supply the final products to the retailers and finally, the retailers are the ultimate places that consumers purchase the products [1]. To analyze the FSC in the current study, the proposed model of Vorst [14] is adopted. According to Vorst [14], the FSC consists of farmers, food processors, distributors, retailers, and consumer handling.

According to the methodology section, 72 documents constitute the database of the current study. According to Table 1, the research objective of 12 out of 72 documents focus on farmers, 21 of them have undertaken research on the food processors, nine documents concentrate on food distributors, 18 documents analyze the retailers in the food industry, four documents concentrate on the consumption and customer handling activities, and eight documents have targeted the entire FSC. The advantage of classifying the documents based on their focus on the supply chain is that it facilitates further analyses on the business models that have been studied in each stage of the FSC.

## 3. Business Model Innovation and Business Model Strategies

The concept of business model provides the opportunity for the entrepreneurs and organizational decision-makers to analyze the logic of their businesses [86]. Indeed, the business model simply explains what values a business creates, to whom, and how it can make money through the value creation and value delivering processes [86]. Many frameworks and models are offered in the literature to analyze a business model, but all the models strive to explain four main aspects of a business: (1) value proposition, which refers to the products and services the business is providing, (2) value delivering, which implies the mechanisms by which the business is connected with its final customers to deliver the products and services to them, (3) value creation, that points out the main activities which are necessary to create and deliver the values to the customers, and (4) value capturing, which indicates the ways a business makes money through the value creation and delivering processes [87].

According to Gambardella and McGahan [88], business model innovation (BMI) is the adoption of novel approaches to commercialize underlying assets. In other words, when a BMI happens the value proposition and the business logic are changed. Amit and Zott [89] believe that BMI occurs in three ways: (1) doing the current business and bonding the current activities in new ways, (2) innovation in the ways the current activities are executed, and (3) formulating new activities. Many driving forces are mentioned in the literature that induces the businesses to innovate their business model. New inventions, human capital, and new technologies are described as the most frequent reasons causing businesses to reconsider their business models [90]. BMI is just not a passive response to the environmental changes, but also it has been considered as a strategy to take advantage of the changes and create competitive advantages for the business [91]. Therefore, the firms in the FSC encounter five strategies to innovate their business model: (1) innovating the value proposition, (2) reconsidering the value delivering mechanisms, (3) innovating the value creation processes, (4) providing new value capturing models, and (5) proposing a quite new business model.

## 4. Methods

**Figure 1:**
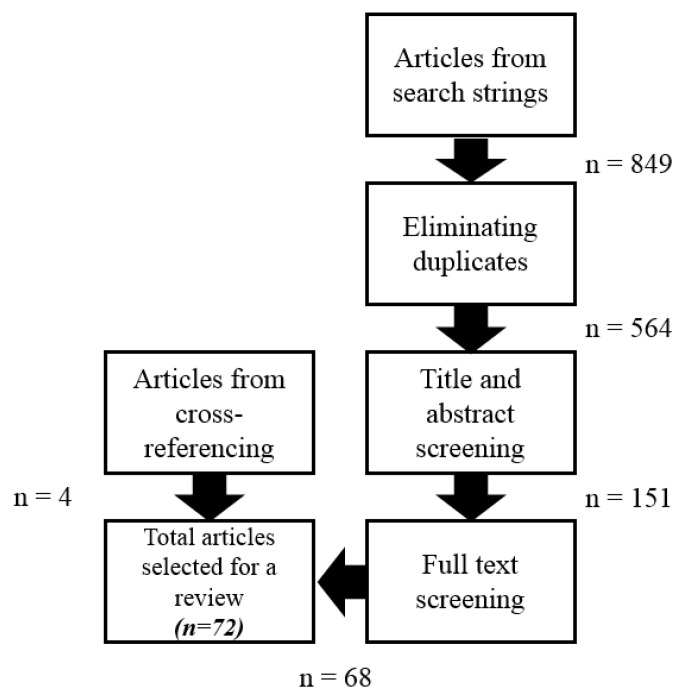
Summary diagram of the systematic selection process.

A systematic literature review on the basis of Prizma methods was conducted to meet the main objective of the current study. Figure 1 depicts the procedure and the steps taken to generate a database of all the relevant published documents that have focused on business models in FSC. The first step was to identify the maximum number of articles published in the common field of the business models and the food supply chain. To do so, firstly, the databases of Thomson Reuters Web-of-Science (WoS), Elsevier Scopus, Science Direct, Emerald Insight, J-store, and Sage Publications, which are the most preferred databases for the research related to the economic and the business management disciplines, were selected for the further consideration. Then, the search query of business model/business models/business model innovation and food (i.e., TITLE-ABS-KEY (“business model*”) AND TITLE-ABS-KEY (food*) and TITLE-ABS-KEY (“business model*”) AND TITLE-ABS-KEY (food*)) for the title, abstract, keywords, or source title were applied in the mentioned databases to identify the published documents in the common area of these two fields. The terms business model and business models were searched separately as some of the databases displayed different results and differentiate these two terms. On the other hand, the word ‘food’ was considered to find the related articles, as most of the articles have not utilized the term of food supply chain directly while they have undertaken research in one of the FSC stages. The combination of search queries maximized the number of published documents in the common field of the business models and the FSC.

**Figure 2:**
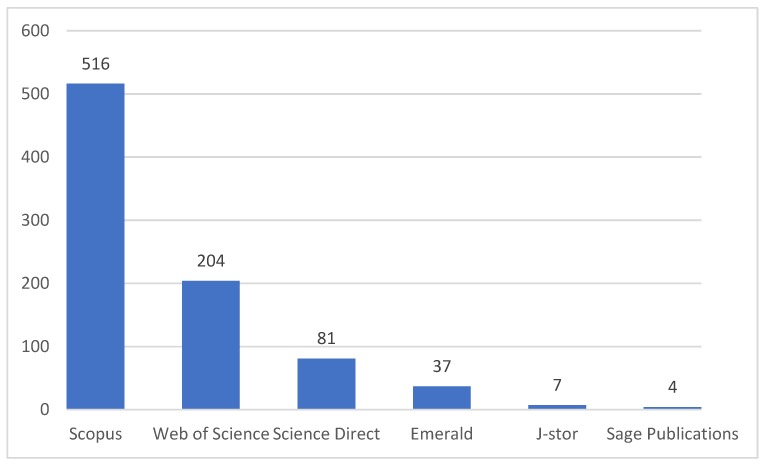
Results of initial search string, last update: November 2019.

In addition, the search was limited to peer-reviewed journals, conferences, and books/book chapters written in English and published in the period of 1999 to November 2019. As it is presented in Figure 1, 849 documents were identified as the result of the initial search. Elsevier Scopus and Thomson Reuters Web of Science, respectively, with 516 and 204 documents (out of 849 found documents) had the most published documents, and the J-Store (with seven documents out of 849 documents) and the Sage publications (with four documents out of 849 documents) had the least share of documents among the other databases considered in this study (see Figure 2).

**Figure 3:**
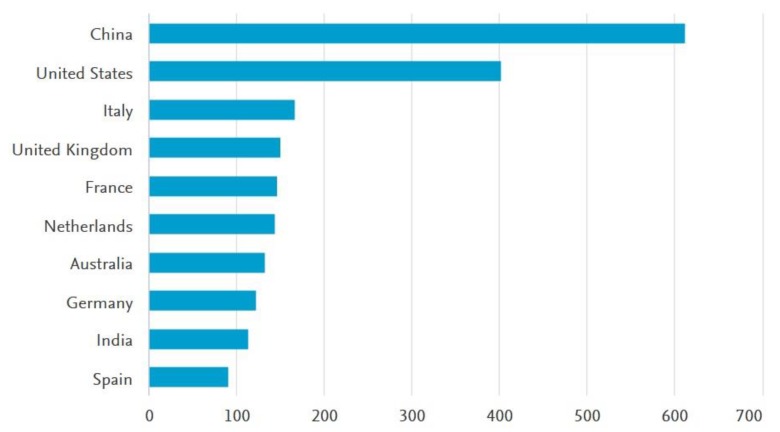
Distribution of publications on business models and food supply chain in different countries.

Articles from the primary search string were categorized by country of origin in which the studies were conducted. It is disclosed that China, as a developing country, has the highest share of the publications on business model innovation (BMI) and FSC. India is also another developing country that has shares of publications in the common area of BMI and FSC. The remaining studies took place in developed countries such as the US, Italy, the UK, France, Netherlands, Australia, Germany, and Spain (see Figure 3).

**Figure 4:**
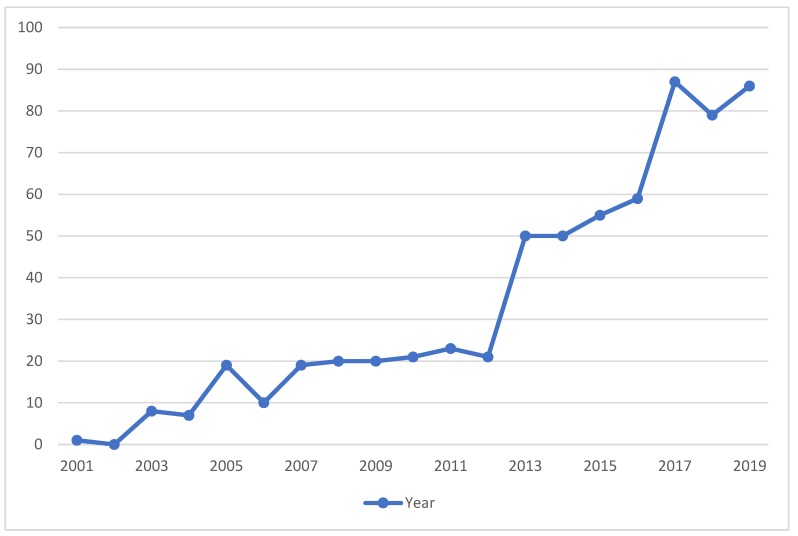
Trends of publications in the common area of the business models and the foods from 1999 to 2019.

According to Figure 1, the second step in selection of final documents was eliminating the duplicated documents which appeared in more than one database during the first step. This step was carried out independently by a two-member panel of authors. In case of debate a consensus, discussion was initiated, involving a third member of authors’ collective. After this step, 564 individual documents were identified. In the next step, the title and the abstracts of these 564 articles was monitored precisely so as to find the relevant documents studied the common area of the business models (BMs) and the FSC. The output of this step resulted in 151 documents ready for the next step. To ensure finding the relevant articles, the full text of 151 documents were studied in detail. As a result, 68 documents were selected for the final analysis as they had all the criteria to meet the objective of this study, since all these 68 articles targeted the common research area of the BMs and the FSC. In addition, four more documents were found to be very suitable for further analysis based on cross-reference checking. Therefore, 72 documents were considered for the final analysis to investigate how the BMI provides solutions to improve the FSC performance. Figure 4 illustrates the trends of the past two decades of publications in the common area of business models and foods.

**Figure 5:**
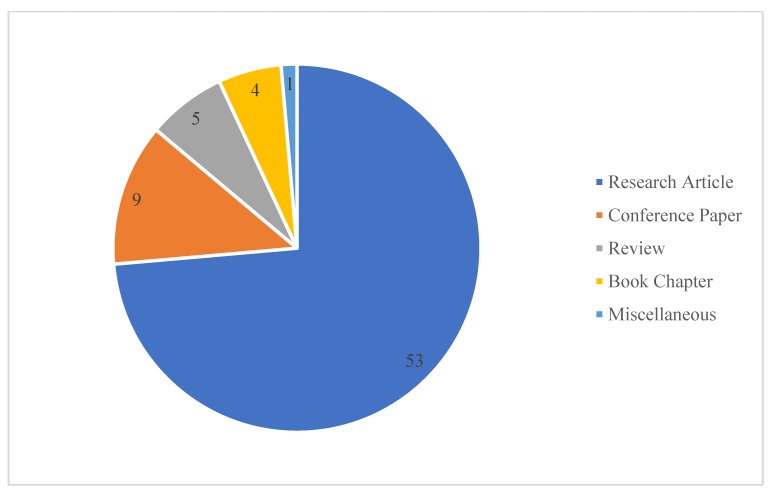
Types of documents considered in the current study.

A close look at the generated database of this study exposed that 53 out of 72 documents were original research articles, nine of them were conference papers, five of them were review articles, four of them were book chapters, and one of the documents was a commentary, published in a peer-reviewed journal (see Figure 5).

In Table 2, the journals with the greatest share of published documents are presented. British Food Journal with the six documents and Journal of Cleaner Production with four documents are journals that published the most articles. According to Table 2, British Food Journal, Journal of Cleaner Production, Sustainability, Business Strategy and the Environment, and Journal of Agriculture Food Systems and Community Development published 23% of the documents (17 out of 72 documents).

The vast majority of the documents (46 out of 72) utilized qualitative empirical research to meet their objective. Quantitative empirical research and conceptual papers with, respectively, 15 and 11 documents were the other research types utilized among the selected documents in this study (see Table 3).

**Figure 6:**
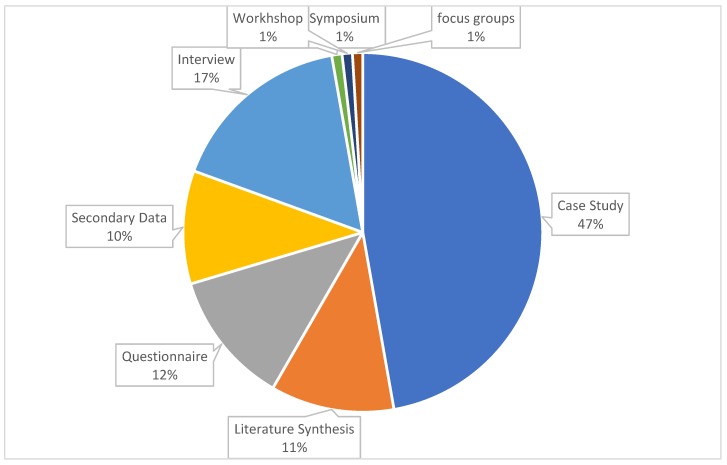
Data collection method of the documents considered in the current study.

The data collection method was the other characteristic checked among the documents and it turned out that most articles collected their data from multiple sources. However, 47% of articles used case studies to collect their data. Literature synthesis, questionnaire administration, secondary data, and interview are, respectively, the other sources of data collection among these 72 articles (see Figure 6). It is worth noting that multiple sources and tools, such as participant observation, focus groups and document analysis, interview, and survey, were used to conduct the case studies to collect data.

In order to provide a better understanding of the analyses presented in the current study, the concepts of food supply chain, business models innovation, and business model strategies are shortly discussed, and then the findings provided.

## 5. Findings

The finding concluded from reviewing the database of the current study reveals that solutions for improving the business model will vary based on the position of a business in the FSC. To analyze the solutions, the documents are categorized based on the part of the FSC they have targeted. In addition, we clarified what business model strategy each paper used to innovate the business model.

### 5.1. Business Model Innovation in the Food Supply Chain

A total of 25 out of 74 reviewed documents in the current study have provided solutions to BMI of the firms and entities of the FSC. Table 4 classifies these 25 documents based on their focus on the FSC and the business model strategies. It means that the documents are firstly classified according to what part of the supply chain is focused on in the article. On the other hand, the position of each document in each row of Table 4 reflects the business model strategy that the document applied to BMI. Each of these articles is described below in detail on the basis of their position in the supply chain.

#### 5.1.1. Farmers

According to Table 4, three of the documents proposed solutions to BMI for the farmers in the FSC in which Pölling, Sroka, and Mergenthaler [16] consider innovation in the value proposition as a solution to BMI for the farmers. While, Varela-Candamio, Calvo, Novo-Corti [26], and Pölling et al. [21] provide a new business model for the farmers in the FSC. The following is a summary of these studies.

##### Value Creation

Pölling et al. [16] elaborate on the importance of city-adjustment in success of urban farming. They sort a set of strategies such as high-value production, direct marketing, and tourism services and they also introduce business models such as ‘low-cost specialization’, ‘differentiation’, and ‘diversification’ for adjusting the farms in the urban areas. Research findings by Pölling et al. [16] resulting from the investigation of 180 urban farms in Ruhr Metropolis, Germany, discloses that the city-adjusted farms reported a better economic performance and anticipated a more positive prospect compared to the non-city-adjusted farms which did not use the mentioned strategies and business models.

##### Business Models

Varela-Candamio et al. [26] propose a conceptual framework to design green business models in which rural women play multiple critical roles in generation-production-consumption of functional foods as producers, educators/advisors, and buyers of such products, where rural women are considered the main educators in the families to boost social-environmental awareness. In the production stage, rural women have a more proactive role in producing functional foods than farmers who are tied with academic institutions for transferring knowledge and producing functional foods. While, in the consumption stage, the role of rural women constitutes demanding functional foods. Moreover, Varela-Candamio et al. [26] debate that functional foods comprise elements added naturally or processed, either to increase human health and well-being or mitigate the risk of diseases.

Utilizing case studies, Pölling et al. [21] develop new solutions to design urban farming business models. According to Pölling et al. [21], farming and agriculture in the urban areas requires unique business models which are distinctive from rural areas. They identify three business models for the urban farming called differentiation, diversification, and low-cost specialization, where the business model of ‘differentiation’ is associated with niche production and differentiation. The differentiation business model recommends that the urban farmers analyze the whole value chain and utilize vicinity to the final consumers and, by a vertical integration, capture more values. Pölling et al. [21] argue that to perform successfully a differentiation business model, not only is integration important but also the product should possess specific features such as exotic species or traditional breeds. Pölling et al. [21] believe that ‘diversification’ in an urban farming business model consists of the variety in the value proposition the farmer offers to the customers. They also articulate that agro-tourism, social events (i.e., education, therapy, health), horse services, and care farming are types of services that urban farms frequently offer to their clients as well. To justify economically urban farming, since the farmland in and around urban areas are smaller than rural areas, higher added values crop production is necessity. Therefore, Pölling et al. [21] explain that the ‘low-cost specialization’ business model is an urban farming model in which only the products with high added values, high transportation costs, freshness, and high perishability are produced because the vicinity to the final consumers is a competitive advantage and makes the model feasible.

#### 5.1.2. Processors

Five out of 25 articles in Table 4 provide solutions for designing and performing the business models for the processors in the FSC where Kähkönen [27] proposes strategies to innovate the value proposition in order to innovate a business model in the food industry and Liberti et al. [30], Giacosa et al. [35], Vojtovic et al. [46], and Jolink and Niesten [39] provide new business models for the processors in the FSC.

##### Value Proposition

Kähkönen [27] studies the concept of value net in the context of the food industry. Kähkönen [27] defines the value net as “a dynamic, flexible network comprising the relationships between its actors who create value through collaboration by combining their unique and value-adding resources, competences and capabilities”. The finding of her study reveals that the value net business model significantly affects the performance of food companies. The finding of the study also illustrates that the actors in the value net in the food industry look for competitive advantages through networking and joint projects. On the other hand, Kähkönen [27] claims that the main aim of value net is to provide value/values to customers through a collaborative process in which shared knowledge leads to innovative and powerful value proposition for food processors.

##### Business Model

Liberti et al. [30] work on an EU funded project called i-REXFO. The main objective of i-REXFO is to design a business model which is able to diminish landfilled food wastes through actions reducing food wastes and producing energy from the inevitable wastes. The i-REXFO model includes four phases. The first phase consists of providing a database to design a tool to analyze the feasibility of the i-REXFO approach in the desired area. The second phase focuses on strategies to minimize the expired food in the retailers. Liberti et al. [30] sort a set of strategies to reduce the expired food such as setting strategic prices and communication policies for pre-expiration food, increasing consumer awareness about food expiration labels, collecting and distributing unsold pre-expired foods to charities, and providing doggy bags among HORECAs (Hotel/Restaurant/Café). The third phase of the i-REXFO business model for avoiding landfilling is to generate energy from expired food in which the food wastes are collected from the retailers and HORECAs and processed to produce biomass biogas plant for electricity production. According to Liberti et al. [30] to test replicability and transferability of the i-REXFO model, this model will be performed in Spain and Hungary.

Giacosa et al. [35] conducted a case study to investigate the approaches to strengthening the business models of family food businesses. They realize that tradition, the family’s values and experiences in the food sector, and innovation, the creation of new values and opportunities by injecting new ideas, are two main pillars that strengthen the business model of a family food business. Giacosa et al. [35] explain that utilizing the customers’ feedback results in product innovation, and it also presents the opportunity to increase the quality and product ranges and, subsequently, it will lead to customer satisfaction. This is because they can offer a wider range of traditional products in old and new flavors utilizing both traditional approaches and the modern technologies. Giacosa et al. [35] provide evidence revealing that considering the tradition and innovation in the family food businesses improve and affect not only the processes of value proposition and value creation but also the models of value delivering and capturing. Vojtovic et al. [46] provide an innovative framework to design a sustainable business model for the processors in the food and beverage industry. Inspired by the business model canvas, Vojtovic et al. [46] propose a ten-pillar business model to develop a sustainable business model for the processors in the food and beverage industry.

They suggest that in the first step, the business concept should be explained where key principles and values that the business offers to the customers, sustainable benefits to the society and the environment, and the company vision and long-term goals should be clearly identified. After explanation of the business concept, the second step is to identify the customers. Vojtovic et al. [46] divide the customers to three categories of early adopters, niche market, and mass segment. The third pillar of this model is building relationships, including branding, habit-forming, and legislation issues. Designing a distribution channel is the next pillar of the proposed sustainable business model of Vojtovic et al. [46]. Vojtovic et al. [46] articulate that planning for resources, designing the key activities (i.e., operating, support, and development) to run the business model, and developing a sophisticated support system are, respectively, the fifth, sixth, and seventh pillars of this model. In this model, developing the partners network with suppliers, manufacturers, service providers is the next action. Estimating the cost structure and selecting the income model are the last two pillars of this model. Jolink and Niesten [39], utilizing the concept of Ecopreneurship, try to develop sustainable business models for the organic food industry. According to Jolink and Niesten [39], ecopreneurs are a subcategory of sustainable entrepreneurs where the business operates in the mass markets and tries to meet the sustainability goals (i.e., economic, environment, and society benefits) at the same time. The result of their study exposes four ecopreneur business models among the organic food companies. The income business model, the subsistence business model, the growth business model, and the speculative model.

Jolink and Niesten [39] argue that the income business model is adopted by small companies whose axial objective is to generate income through creating the opportunity for consumers to eat healthy foods. Providing the proper information to the consumers about eco-products plays a critical role in this model. In accordance with the findings of Jolink and Niesten [39], the objective of the companies applying the subsistence business model “… is to survive and meet basic financial obligations”. Although they try to make the world better, being ecologically sustainable is not their priority, since they need to reach the mass markets, and lack of sufficient organic raw materials restricts them to present eco-product to their customers. Therefore, they need to make a compromise between being economically and environmentally sustainable. The third ecopreneur business model discovered among the organic food companies is the growth model, where the focal point is to invest and reinvest on the financial aspects and the relationship with the customers in order to be profitable in the long term. According to Jolink and Niesten [39], those companies that implement such a business model have a relatively large impact on the market. These companies have turned being sustainable to a competitive advantage and have become profitable in this way. Jolink and Niesten [39] express that the speculative model is the fourth ecopreneur business model they identified among the organic food companies. According to Jolink and Niesten [39], the speculative model focuses on making money by selling eco-products where the economic profits are set in the priority. Indeed, in this model, sustainability is turned into a tool for profitability. These ecopreneurs concentrate on short-term goals

#### 5.1.3. Distributors

Among the research documents reviewed in the current study, four of them study business models of food distributors in which Shih and Wang [48] and Kim et al. [54] investigate new solutions for delivering the food productions to the customers and Berti et al. [50] and Martikainen et al. [55] introduce new business models for the distributors in the FSC.

##### Value Delivering

One of the most important issues in the FSC is food distribution, where cold chain management plays a vital role. Having a frozen storage with the risk of high-energy consumption and cool storage with the threat of bacterial decay is a dilemma the distributors in the food industry deal with. Hence, Shih and Wang [48] by means of an Internet-of-Things (IoT) architecture and ISO 22,000, an international food standard, propose four solutions to overcome the aforementioned problems in the food distribution: cold chain home delivery service, convenience store (CVS) indirect delivery, CVS direct delivery, and flight kitchen service. According to their results, applying the above-mentioned business models could result in a 1.36 million increase in annual sales of braised pork rice, generating extra revenue of US$6.35 million by creating new distribution channels, and also reducing 10% energy consumption.

Shih and Wang [48] elaborate that cold chain home-delivery service refers to free home delivery of the foods in 1–2 working days for orders exceeding a minimum purchase requirement at the off-peak hours (14:00–17:00). This approach not only provides the opportunity to use the cold storage less, but also expands brand recognition and facilitates market penetration. On the other hand, Shih and Wang [48] express that CVS indirect delivery refers to delivering fresh food products that are processed in original equipment manufacturing (OEM) facilities by CVS companies. Convenience store companies prefer cool storage products than the products needed to be thawed where reheating them does not take more than 30–40 s in a microwave. In addition, Shih and Wang [48] argue that CVS direct delivery refers to the food products are processed, packed, and delivered by CVS. This approach is selected in the case where food quality and food safety are very important. According to Shih and Wang [48], the flight kitchen business model is quite similar to the CVS indirect delivery business model where the only difference is the lower supply volume and fewer supply spots. In accordance with the flight kitchen business model, semi-processed food products are delivered to international catering companies via cool storage. Then they process and deliver it to the airplane flight kitchen, where they just need to re-heat it. In this approach, daily delivery is very important to maintain food safety and quality. To solve the urban agriculture’s problems, Kim et al. [54] propose the Eco-M business model where organic fresh food produced by suburban agriculture is delivered daily to the local markets. Kim et al. [54] claim that although this model has performed successfully, it cannot benefit from competitive price since the risk of wasted food is high as the products are fresh foods and their expiration date is too close to the production date, and they should be consumed in 10 days after production.

##### Business Model

Berti et al. [50] propose a disruptive business model producing new values and new markets by redefining the food supply chain. They introduce a digital food hub, an online marketplace, facilitating efficient connections among local food producers and consumers. Berti et al. [50] argue that it is a sustainable business model as it increases the demand for the local food, and it also promotes healthy and sustainable food for the local communities. Berti et al. [50] believe that this digital food hub, indeed, provides a strategic network across the food supply chain to coproduce socioenvironmental shared values.

Martikainen et al. [55] try to design business models for third-party logistics service providers (LSP) for local food supply chains. Utilizing the business model canvas, they propose two new business models named business model for the focused service offering and business model for the full-service offering. The main difference between the focused and full-service offerings is the market that they have targeted. In the focused business model, the concentration is on upstream producers and processors, while the full offering covers downstream operators’ needs. Therefore, this difference in the target market reflects on a different value proposition and, subsequently, a different business model. Table 5 provides the opportunity to compare two business models by elaborating the models in detail.

#### 5.1.4. Retailers

The retailers play a very important role in the FSC as these are the places that the food products are delivered to the final customers. This part of the FSC attracted more researchers as eight out 25 documents have focused on different aspects of the business model of the retailers. Di Gregorio [56] propose strategies for the value proposition the retailers are delivering, Huang et al. [62] focus on how retailers can create values, Kaur and Kaur [64] and Pereira et al. [69] study innovative solutions for retailers to innovate their business model by redesigning value delivering models. Finally, Cheah, Ho, and Li [73], Franceschelli et al. [61], Ribeiro et al. [70], and Lu et al. [66] identify new business models for the retailers in the FSC. The following is a summary of all these studies.

##### Value Proposition

Di Gregorio [56] proposes a creative business model for retailers, where the products are delivered to the customers, in the food industry. He applies the concept of a placed-based business model to introduce a model in which location-specific resources are used to create and capture value. Di Gregorio [56] conducted case studies within the slow food industry in Italy (Coop Italia and Eataly). According to his results, the place-based business model in the slow food industry in Italy will subsequently lead to resilience, sustainability, and prosperity of the social context by reviving passion for traditional food cultures and increasing supply and demand for local food products.

##### Value Creation

Huang et al. [62] develop a e-business model for the food souvenir industry in Taiwan, inspired by the e-commerce business model. The focus of their innovation is value creation. According to their model, the final customers are able to order online, and local providers are responsible for the supply and delivery of orders.

##### Value Delivering

Utilizing a sensor-based measurement containers (SBMCs), an Android application, and cloud IoT-enabled grocery management system (CE-GMS), Kaur and Kaur [64] provide a creative solution to business model innovation for retailers in the FSC. By designing an innovative approach to get the order and deliver it to the customers, they have created a new business model. According to proposed model of Kaur and Kaur [64], when the retailers get the order from a customer, they subsequently get an alarm related to the quantity of the product in the store and the warehouses at the same time. This contribution helps them to manage the quantity of the products and make them able to maximize their potential to cover the customers’ needs.

Pereira et al. [69] ran a case study to investigate a sustainable business model for delivering fresh milk. Pereira et al. [69] compared traditional channels and vending machines for supplying the fresh milk. Their finding discloses that utilizing vending machines shortens the supply chain; therefore, it has a lower impact on the environment due to the elimination of mediators and transportations activities. Pereira et al. [69] also realized that the success of vending machines remarkably depends on consumer behavior. Their findings revealed that when the consumption of environmentally friendly products is very important to the consumers the vending machines were more profitable.

##### Business Model

Cheah et al. [73] provide empirical evidence that business model innovation provides competitive advantages to the retailers in the food industries. Their finding illustrates that the retailers acting in a high turbulence environment have a higher chance to get sustainable competitive advantages by re-innovation of their business model. Franceschelli et al. [61] propose a framework to design a sustainable business model for a food startup in which, in addition to the economic profit, the social and environmental benefits are considered. Their study focused on a pizzeria startup in Italy.

Ribeiro et al. [70] strive to test the sustainability of a retailing strategy so-called ugly business model in which the waste from fresh fruits and vegetables that are not sold through the conventional distribution channels, due to the appearance of these products, is minimized. According to their results, this project, in addition to the economic benefits, has had social benefits (i.e., “increasing waste awareness and healthy food consumption and community engagement in reduction of the waste”, etc.) and environmental benefits (i.e., “prevent food wastes and climate change mitigation benefits”).

Lu et al. [66] provide solutions for designing a business model for sustainable agricultural products utilizing Internet-of-Things (IoT). Aided by IoT, the new business model provides products through networks and e-commerce via electronic data interchange and e-mail online sales contract along with the traditional marketing channels. The convenience of online shopping and instant messaging interoperability are mentioned as new value propositions that IoT can offer for the sustainable agriculture. Lu et al. [66] also claim that IoT designs a sophisticated information system in the organizations which arms the businesses to design a customer-centric structure collecting the data and customers’ feedbacks and also provides adequate information to the customers.

#### 5.1.5. Consumers

Consumers are the most important part of the FSC, as, without the consumers, all the supply chain will be meaningless. Handling customers’ issues and studying their behavior is of the utmost importance during managing the FSC. Martinovski [74] has an innovative approach to design value propositions based on the customers’ behavior.

##### Value Proposition

Martinovski [74] has a different perspective to design a business model for an entity performing in the FSC. Martinovski [74] believes that consumer behavior is the key determinant in designing a business model. Therefore, he proposes the concept of modeling a business model according to consumer behaviors while purchasing food products. His finding reveals that this approach is a tool for the decision-makers to design a sustainable business model in which, on the one hand, the businesses are able to utilize this customer-centric approach to get the customers’ feedback for developing corresponding value propositions for their target market and on the other hand, society and customer benefits are considered and healthy safe food productions are delivered to them based on their feedback.

#### 5.1.6. The Entire Supply Chain

In addition to the studies that targeted a specific stage of the FSC, many studies have focused on the whole supply chain and have provided solutions to create values for the whole FSC. Designing solutions for value creation and value delivering for the entire supply chain, indeed, implies that the researchers have tried to provide innovative solutions that affect the whole supply chain from the farmers to the retailers and customers. For instance, Adekunle et al. [78], Barth et al. [79], Ulvenblad et al. [84], and Pakh and Baek [82] recommend frameworks to innovate the business models in the FSC.

##### Business Model

Adekunle et al. [78] designed a business model for the small millets value chain in India. According to their result, a mixed customer intimacy-product leadership (CI–PL) business model is appropriate for the small millets value chain in India where the CI-business model refers to customer intimacy business models in which the customer is placed in the center of the business model and PL business model highlights the product leadership business models in which the quality of the product is of the utmost importance. According to the proposed business model of Adekunle et al. [78], there should be an interactive collaboration among farmers, technologists, processors, and researchers to produce and deliver high-quality small millets through innovation, creating, and sharing knowledge. This collaboration will lead to increase yield, improved marketing, and reduction of drudgery. Barth et al. [79] developed an approach to design an innovative sustainable business model for the businesses performing in the agri-food sector statements. Based on a deep literature review, they designed questions for development of each pillar of the business model. According to Barth et al. [79] the business model constitutes four main pillars of (1) value proposition, (2) value creation and delivery, (3) value capture, and (4) value intention. In Table 6 their proposed business model is presented.

Ulvenblad et al. [84] studied the barriers to business model innovation in the agri-food industry. To do so, they ran a systematic literature review where they reviewed 570 research articles published between 1990 to 2014. They, ultimately, categorize the barriers to BMI in the agri-food industry into two classes of internal barriers and external barriers, where the internal barriers to BMI include (1) individual barriers (e.g., perceptions, values, behavior), (2) organizational barriers (e.g., lack of competencies, insufficient resources, and unsupportive organizational structure). On the other hand, Ulvenblad et al. [84] articulate that the external barriers to BMI comprise (1) resistance and lack of support from specific actors and (2) restrictive macro-environment. It is worth mentioning that they provide another layer of analysis for these barriers, and for each of the mentioned barriers, they provide sub-variables.

Pakh and Baek [82] develop the concept of ‘considerate design approach’ to design a sustainable business model in which value propositions have considered meeting the benefits of all the stakeholders. Pakh and Baek [82] develop four business models including (1) neighboring producer community, a collaboration platform between the local farmers/producers and the customers for direct sale, (2) local food café, a mediator between local farmers/producers and the customers where the local foods are served, (3) farm mentoring institute, a mentoring platform transferring the farmers’ knowledge to the others and students, and finally (4) food community, including cuisine researchers and educators training the locals to utilize local ingredients to cook professionally in order for either their own consumption or selling of their food.

## 6. Discussion

To increase food supply, many solutions are provided in the literature in which 72 published documents have studied the business models of the businesses in the FSC. A deep analysis of these documents illustrates that 25 out of 72 documents present strategies and solutions to innovate the business models in the FSC for improving the performance of the FSC. Three of these articles provide recommendations to redesign the value propositions in the business models. Kähkönen [27] introduces the concept of value net, which suggests collaboration among the different stakeholders to shape the value. Martinovski [74] also has a similar recommendation to design the value proposition. He recommends engaging the customers in the value shaping processes. While Di Gregorio [56] has an innovative solution for the value proposition as he considers the location and the place as a source of value.

**Figure 7:**
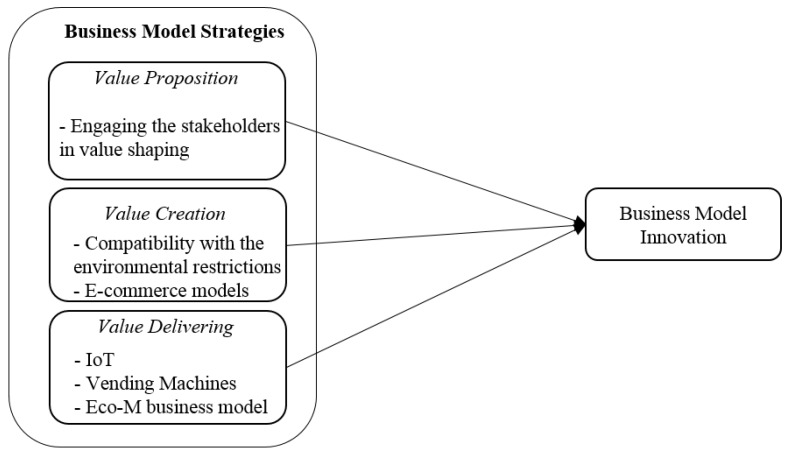
Business model strategies to business model innovation in the FSC based on the literature.

Two of the documents, including Pölling et al. [16] and Huang et al. [62], consider innovation in the value creation processes as the strategy to BMI for the food industry. Pölling et al. [16] sort out solutions to adjust the urban farms according to the cities’ constraints. On the other hand, Huang et al. [62] provide empirical evidence proving that applying e-commerce models facilitates the value creation processes. Reconsidering the value delivery processes is another strategy considered by the author to BMI in the FSC. Shih and Wang [48] and Kaur and Kaur [64] recommend applying IoT to optimize the management of delivering the food production. Pereira et al. [69] offer vending machines to deliver fresh milk products in the urban areas and Kim et al. [54] introduce the concept of Eco-M business model to facilitate delivering fresh foods to urban areas (see Figure 7). These strategies are summarized in Figure 6 as the strategies are applied in the FSC to innovate the business model.

**Figure 8:**
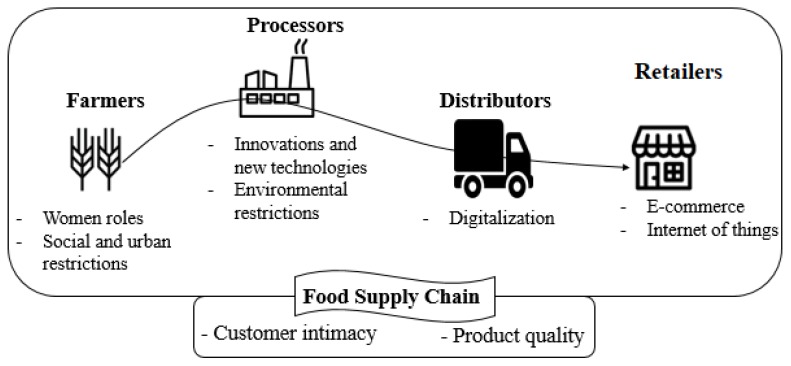
The business model innovation driving forces in the food supply chain.

Along with the mentioned studies, there are studies that present BMI driving forces either for a specific part of the FSC or for the whole of the FSC. For instance, Varela-Candamio et al. [26] claim that women not only play vital roles in designing and implementing a sustainable business models in the farms but also raise the demand for sustainable products by increasing awareness of local food in communities. Pölling et al. [21] argue that urban farms should adapt their business model according to the conditions of the city. Giacosa et al. [35] see adding innovations and technologies to traditional mechanisms as a source of innovation in business models for the food processors as proposed in [93,94]. Besides, Liberti et al. [30], inspired by the circular economy concept and circular business models, provide recommendations to produce energy from the inevitable wastes. Berti et al. [50] disrupt the current FSC, affected by digitalization. They introduce a digital food hub, which is an online marketplace, to connect the local food producers and consumers. Ultimately, Adekunle et al. [78] consider the customers intimacy and the product quality as driving sources to BMI for the whole FSC (see Figure 8).

## 7. Conclusions

Food security is a very important issue for both researchers and practitioners struggling to provide solutions for supplying adequate foods to the next generations. Various remedies and recommendations are given in the literature to bridge the gap between food supply and food demand for the next 50 years, including BMI. BMI is a tool allowing the firms in the FSC to optimize values they are creating and delivering to their customers. By means of a systematic literature review, the current study unfolded that the strategies such as engaging the stakeholders in value creation processes, compatibility with the social and environmental constraints, utilizing e-commerce models, and ultimately, applying IoT, vending machines, and eco-business models for delivering products to customers are considered by the literature to BMI in the FSC. In addition, one of the contributions of the current study was to distinguish the driving forces of BMI based on the position of the firms in the FSC where the literature illustrates that rural women and social and urban conditions are the most important driving forces inducing the farmers to reconsider their business model. Besides, it is disclosed that inventions and new technologies, and environmental issues are the main driving forces to BMI for the food processors. Digitalization has disruptively changed the food distributors models. E-commerce models and Internet-of-Things have been the factors causing retailers to innovate their business models. It was also found that customer’ needs and product quality are two main factors affecting the business model of all the firms operating in the FSC regardless of their position in the chain. At the same time, the behaviors of consumers is changing radically too. Further investigations necessitate a complex approach, focusing on consumer-producer interactions, taking into consideration the changing material and digital environment.

On the one hand, the findings of the current study provide a fundamental insight for the mangers and the entrepreneurs of the food industry to design a business model which has the ability to predict and adapt to environmental changes and also is able to improve the performance of the FSC. On the other hand, these findings can be a basis for future research. It is recommended to the researchers to design business models for each of the players of the food supply chain based on the findings of this study.

## Figures and Tables

**Table 1:** Categorizing the reviewed articles based on their focus on the food supply chain (FSC).

Explanation	Farmer	Processor	Distributor	Retailer	Consumer	The Whole Value Chain
Sources	Blasi, et al. [15]; Pölling, et al. [16]; Krivak, et al. [17]; Hooks, et al. [18]; Morris, et al. [19]; Panța [20]; Pölling, et al. [21]; Robinson, et al. [22]; Siame [23]; Tushar, et al. [24]; van Eijck, et al. [25]; Varela-Candamio [26].	Kähkönen [27]; Lange and Meyer [28]; Zucchella and Previtali [29]; Liberti, et al. [30]; Bhaskaran, and Jenkins [31]; Bogers and Jensen [32]; De Bernardi, and Tirabeni [33]; Di Matteo and Cavuta [34]; Giacosa [35]; Harringon and Herzog [36]; Hemphill [37]; Hutchinson, et al. [38]; Jolink, and Niesten [39]; Jia, et al. [40], Markowska, et al. [41]; Mars [42]; Morris, et al. [43]; Sardana [44]; Svensson and Wagner [45]; Vojtovic, et al. [46].	Samuel, et al. [47]; Shih, and Wang [48]; Wubben, et al. [49]; Berti, et al. [50]; Bruzzone, et al. [51]; Gitler [52]; Hong, et al. [53]; Kim, et al. [54]; Martikainen, et al. [55].	Di Gregorio [56]; Chang, Wei, and Shih [57]; Dawson [58]; Fiore, et al. [59]; Franceschelli and Santoro [60]; Franceschelli, et al. [61]; Huang, et al. [62]; Karpyn, et al. [63]; Kaur, and Kaur [64]; Lin, L., et al. [65]; Lu, et al. [66]; Massa and Testa [67]; Morris, et al. [68]; Pereira, et al. [69]; Ribeiro, et al. [70]; Russell and Heidkamp [71]; Sebastiani, et al. [72]; Cheah [73].	Martinovski [74]; Ukolov, et al. [75]; Balcarová, et al. [76]; Franchetti [77].	Adekunle, et al. [78]; Barth, et al. [79]; Long, et al. [80]; Minarelli, et al. [81]; Pahk and Baek [82]; Vivek [83]; Ulvenblad, et al. [84]; Zondag, et al. [85].
Numbers	12	21	9	18	4	8

**Table 2:** Journals with the largest number of documents.

Journal Name	Number of Documents
British Food Journal	6
Journal of Cleaner Production	4
Sustainability	3
Business Strategy and the Environment	2
Journal of Agriculture Food Systems and Community Development	2
Total	17

**Table 3:** Documents based on research type.

Explanation	Conceptual	Qualitative Empirical	Quantitative Empirical
Sources	Lange and Meyer [28]; Zucchella and Previtali [29]; Liberti, et al. [30]; Berti, Mulligan, and Yap [50]; Barth, Ulvenblad, and Ulvenblad [79]; Huang, Lee, and Lee [62]; Kim, Lee, and Yang [54]; Lu, et al. [66]; Panța [20]; Soundarrajan and Vivek [83]; Ulvenblad, et al. [84].	Di Gregorio [56]; Kähkönen [27]; Samuel, Shah, and Sahay [47]; Ukolov, et al. [75]; Krivak, et al. [17]; Hooks, et al. [18]; Bhaskaran, and Jenkins [31]; Adekunle, et al. [78]; Bruzzone, et al. [51]; Chang, Wei, and Shih [57]; Dawson [58]; De Bernardi, and Tirabeni [33]; Fiore, Conte, and Conto [59]; Franceschelli and Santoro [60]; Franceschelli, Santoro, and Candelo [61]; Franchetti [77]; Giacosa, Ferraris, and Monge [35]; Gitler [52]; Harringon and Herzog [36]; Hemphill [37]; Hutchinson, Singh, and Walker [38]; Jolink, and Niesten [39]; Karpyn, and Burton-Laurison [63]; Kaur, and Kaur [64]; Lin, L., et al. [65]; Long, Looijen, and Blok [80]; Markowska, Saemundsson, and Wiklund [41]; Mars [42]; Martikainen, Niemi, and Pekkanen [55]; Massa and Testa [67]; Morris, Jorgenson, and Snellings [19]; Ogawara, Chen, and Zhang [68]; Pahk and Baek [82]; Pereira, et al. [69]; Pölling, et al. [21]; Ribeiro, et al. [70]; Robinson, Cloutier, and Eakin [22]; Russell and Heidkamp [71]; Sardana [44]; Sebastiani, Montagnini, and Dalli [72]; Siame [23]; Svensson and Wagner [45]; Tushar, et al. [24]; Ulvenblad, Ulvenblad, and Tell [92]; Vojtovic, Navickas, and Gruzauskas, [46]; Zondag, Mueller, and Ferrin [85].	Blasi, Ruini, And Monotti [15]; Martinovski [74]; Shih, and Wang [48]; Wubben, Fondse, and Pascucci [49]; Pölling, Sroka, and Mergenthaler [16]; Balcarová, et al. [76]; Bogers and Jensen [32]; Cheah, Ho, and Li [73]; Di Matteo and Cavuta [34]; Hong, et al. [53]; Jia, et al. [40]; Minarelli, Raggi, and Viaggi [81]; Morris, Shirokova, and Shatalov [43]; van Eijck, et al. [25]; Varela-Candamio, Calvo, and Novo-Corti [26].
Number	11	46	15

**Table 4:** Business model innovation and supply chain and business model strategies.

Explanation	Farmers	Processors	Distributors	Retailers	Consumers	The Entire Supply Chain	Numbers
Value Proposition		Kähkönen [2]		Di Gregorio [3]	Martinovski [4]		3
Value Creation	Pölling, Sroka, and Mergenthaler [5]			Huang, Lee, and Lee [6]			2
Value Delivering			Shih and Wang [7], Kim, Lee, and Yang [8]	Kaur and Kaur [9], Pereira et al. [10]			4
Value Capturing							0
Business Model	Pölling et al. [11], Varela-Candamio, Calvo, Novo-Corti [12]	Liberti et al. [28], Vojtovic, Navickas, and Gruzauskas [29], Giacosa, Ferraris, and Monge [30], Jolink and Niesten [50]	Berti, Mulligan and Yap [56], Martikainen, Niemi, and Pekkanen [79]	Cheah, Ho, and Li [62], Franceschelli, Santoro, and Candelo [54], Ribeiro, Sobral, Peças, Henriques [66], Lu et al. [20]		Adekunle et al. [83], Barth, Ulvenblad and Ulvenblad [84], Pakh and Baek [56], Ulvenblad et al. [27]	16
Numbers	3	5	4	8	1	4	25

**Table 5:** The difference between focused and full-service offering business models.

Business Model Components	Focused to Service Offering	Full Service Offering
Value Proposition	Improvement of customer’s ability to satisfy the service needs of their customers and cut the costs	Being and interface in production and consumption chain in a cost-effective way
Key customers	Food producers	Food producers and trade, especially wholesalers, retailers and the HORECA sector
Customer Relationships	Daily routine, based on traditional ways of communication	Communication strategies and forms are highly individualised and based on actual situations as opposed to formerly defined algorithms
Channels	Long term contracts and agreements	Long term contracts as well as short term service agreements
Key Activities	Facilitation of logistical and financial processes	Facilitation of logistical and financial processes and active participation in marketing and selling of different products, product quality management
Key Resources	Logistical knowledge and infrastructure	Logistical knowledge and infrastructure and in-depth knowledge of product and market conditions
Cost Structure	Transaction-based agreements with partners	ransaction-based agreements with partners—efficient consumer response systems
Revenue Streams	Revenues are determined by volume, compensation on flexibility and increasing of volume	Revenues are tied to joint value creation along the food chain

Source: own construction based on Martikainen et al. [55].

**Table 6:** Proposed business model of Barth et al. [79] for the agri-food sector.

Pillars	Degree of Innovation	Sustainability
Value proposition	Innovation in product/services Innovation in market	Sustainability of products/services
Value creation and delivery	Innovation in channels Innovation in partner network Innovation in technologies	Sustainability of creation and delivering product/services processes
Value capture	Innovation in cost structure innovation in reveue streams	Sustainability of revene streams and cost structure
Value intention	Innovative attitudes	Sustainability of the goals
